# A reliable measure of similarity based on dependency for short time series: an application to gene expression networks

**DOI:** 10.1186/1471-2105-10-270

**Published:** 2009-08-28

**Authors:** Mônica G Campiteli, Frederico M Soriani, Iran Malavazi, Osame Kinouchi, Carlos AB Pereira, Gustavo H Goldman

**Affiliations:** 1 Departamento de Ciencias Farmacêuticas – Faculdade de Ciências Farmacêuticas de Ribeirão Preto Universidade de São Paulo, Ribeirão Preto, SP, Brazil; 2 Departamento de Física e Matemática – Faculdade de Filosofia, Ciências e Letras de Ribeirão Preto Universidade de São Paulo, Ribeirão Preto, SP, Brazil; 3 Instituto de Matemática e Estatíistica Universidade de Sao Paulo, São Paulo, SP, Brazil

## Abstract

**Background:**

Microarray techniques have become an important tool to the investigation of genetic relationships and the assignment of different phenotypes. Since microarrays are still very expensive, most of the experiments are performed with small samples. This paper introduces a method to quantify dependency between data series composed of few sample points. The method is used to construct gene co-expression subnetworks of highly significant edges.

**Results:**

The results shown here are for an adapted subset of a *Saccharomyces cerevisiae *gene expression data set with low temporal resolution and poor statistics. The method reveals common transcription factors with a high confidence level and allows the construction of subnetworks with high biological relevance that reveals characteristic features of the processes driving the organism adaptations to specific environmental conditions.

**Conclusion:**

Our method allows a reliable and sophisticated analysis of microarray data even under severe constraints. The utilization of systems biology improves the biologists ability to elucidate the mechanisms underlying celular processes and to formulate new hypotheses.

## Background

In recent years, the technology of DNA microarrays has been central for the knowledge assembly in molecular biology research. The possibility of measuring mRNA levels in a genomic scale in a comparative way enables the discovery of genes or clusters of genes which expressions are differentiated in a specific condition, thus providing important clues about gene functions and pathways. Statistical tools have been developed for the analysis of this great amount of biological data aiming to help biologists to make predictions and originate hypotheses to be experimentally tested.

In the past decade, systems biology approaches are emerging as a novel concept increasingly attractive to deal with this kind of data. The biological system is modeled as a graph and each biological unit (a gene, for instance) is represented as a node in this graph and if two nodes interact they are connected by an edge [1,2]. This approach produces a visually appealing structure generically called network where nodes with similar characteristics can be grouped together and groups of tightly interconnected nodes are often associated with a specific cellular function [3,4]. Perhaps the most interesting feature of this application is the possibility to analyze how these modules of tightly interconnected nodes connect among themselves or how different functions interact to produce the observed phenotype. In fact, this kind of approach is proving to be a more robust tool for the extraction of relevant information from the biological data and abstraction and better comprehension of the biological system as a whole [5].

In the specific case of networks inferred from gene expression data, an interaction between two genes can be determined by a measure of association between their expression patterns. Thus, in order to get a reliable structure, one must first define a consistent measure to quantify gene expression similarity. The most commonly ones used are based on Pearson correlation (for linear association) [6,7] or mutual information (that accounts for any kind of association function) [8-10]. These are very robust measures and are widely used in a number of applications involving the inference about the degree of dependency between biological signals.

However they are based on statistical assumptions that depends on assymptotic supositions making them strongly dependent on large samples sizes. This becomes an important drawback in the context of molecular biology applications. DNA microarrays are still a very expensive technique and the design of experiments with a large number of different conditions and/or time acquisition demands such human and financial resources that could make it unachievable in many research laboratories. Thus, the usual procedure is to design experiments with a small number of observations. In fact, according to the Gene Expression Omnibus [11], about one third of microarray studies involves experiments with 3–8 time points or other types of non-temporal sequential data [12].

Limited sampling accentuates the difficulties related to standard signal analyses. Among the most important problems are the influence of noise that becomes even more prominent with shorter series, enhancing the complexity in distinguishing real from random patterns and increasing the potential of misleading results [13]. The efforts to overcome difficulties related to limited sampling include strategies of simplification [14,15] and the incorporation of multi-source information [16]. In the first case, the goal is to transform continuous data to discrete representations prior to analysis, categorizing the gene expression data into a set of diffierent states trying to capture tendencies instead of absolute values. However, simplification strategies are highly dependent on pre-definitions about the *a priori *patterns of gene expression in the discretization step, a process which is largely dependent on the researchers' expertise [13]. Additionaly, valuable information is lost during this process. On the other hand, incorporating multi-source information includes prior knowledge or multi-scale and different levels of information from other sources to improve the computational analysis of short time-series microarray data. This approach faces the challenge of dealing with a high heterogeneity of data which increases the difficulties in extracting meaningful information. Furthermore, for many organisms for which the state of research is still in its infancy, there is no reasonable additional information or any prior knowledge to rely on. Notwithstanding the statistical difficulties inherent to this kind of analysis, these data represent a rich source of information and the design of a rational pipeline to better explore them is of paramount importance.

Here we present a method of inference of dependency between series from a temporally poor data set and apply this method to the construction of condition-specific subnetworks from gene expression data. The proposed method is an alternative to the standard measures of dependency. It performs well with short series and requires no *a priori *assumptions. In order to evaluate the method, we show results for an adapted subset of a benchmark data set of *Saccharomyces cerevisiae *[17] aiming to mimic the limiting conditions above described. We show that the method is efficient in capturing dependencies between the gene signals and that these dependencies can be related to the existence of a common transcription regulatory factor with a high confidence level. Our results strongly suggest that the method proposed allows the application of systems biology to data sets obtained from limited amount of experiments and that this method offers the biologist a robust tool to analyze gene expression data.

### A similarity measure

The output of a microarray experiment is given as the log of the ratio between the amount of mRNA of an experimental sample and a control sample. This ratio is commonly called log-ratio and gives the degree of modulation of a gene relative to the control sample. A common attempt in these experiments is to compare networks originated from different experimental conditions trying to find either similarities or differences that explain the observed phenotype. For example, one could create different mutant strains inactivating key genes and then analyze separately the expression profiles of each mutant in comparison to the expression profiles of the wild type. This approach would generate different networks promptly revealing the distinct adjustments the organism takes in response to each experimental condition.

The present work aims to reconstruct the genes co-expression networks in a condition specific way. To make this reconstruction possible, we introduce a measure of association between gene profiles (log-ratios measurements). Consider, thus a data set consisting of log-ratios for *N *genes in *ρ *different experimental conditions. The parameter *ρ *accounts for different series of experiments hence, different physiological conditions as well as different time points. Consider also that we are particularly interested in a specific subset of *ρ *say, a specific series of experiments consisting of *ρ' *sample points. The question addressed here is how to infer similarity between two gene profiles consisting of *ρ' *sample points, considering usually *ρ' *< 10. One can think of some points worth to consider when idealizing an efficient method:

1. The function that relates co-regulated genes is not necessarily linear. In fact it can assume very complex non-linear forms. Thus, an efficient method of inference of gene relations should be able to capture any association function that should arise naturally rather than being inforced *a priori*;

2. Co-regulated genes are expected to respond in a correlated way in different experimental conditions and even the lack of modulation in a given experimental condition is actually an useful information. Although interested in constructing condition-specific networks, we would like to use the valuable information contained in other possible series of experiments to determine the associated pairs. Thus, information about how a given pair of genes behaves in all known *ρ *conditions could be taken into account when calculating a measure of similarity between pairs of gene expression profiles;

3. Microarray experiments involve several factors where chance fluctuations and random processes play a significant role. Thus, points around zero, or very small modulation rates are more likely to reflect experimental artifacts and lack biological meaning than higher values. It would thus be desirable if the method gave different weights to different levels of modulation;

Based on these leads, the similarity *S*^*ij *^between genes *i *and *j *concerning *ρ' *conditions can be defined as:

(1)

where  is the modulation rate of gene *i *in condition *r*. Notice that the score *S *can be obtained summing over any subset of *ρ *allowing the researcher the choice of analysis of each perturbation individually or the entire data set depending on his/her interests.

Define now a random vector (*X*; *Y*) whose elements are modulation rates of two genes to be observed in one experimental condition. Considering (*x*; *y*) as a possible observation of (*X*; *Y*), the function *s *as used in this work is defined as follows:

(2)

for *f *and *g *being, respectively, the marginal densities of *X *and *Y *and *h *the joint probability density of (*X*; *Y*). Equation 2 is known as a log-likelihood score in the context of testing independence of two random variables. We do not know the form of these densities *a priori*. In this work we use the data information contained in all *ρ *experiments to estimate it. Note that if we have *h *we simply derive the marginal densities *f *and *g*. In the sequel we delineate the way *h *is estimated.

In order to estimate *h*, we compile a list of co-occurrences for the *N *genes in the *ρ *experimental conditions. This list is obtained by taking all the possible pairwise combinations of modulation rates for each experimental condition. The number of pairs is . Note that the set of *N *genes is previously filtered to comprise only the genes modulated in at least one experimental point. This step reduces largely the computational cost. With the co-occurrences list one can construct a bi-dimensional frequency matrix from which is estimated the joint probability density in equation 2. The rationale behind this is that, in an ideal situation all the genes modulated in a specific condition are somehow related to the perturbation(s) impinged to the system and, thus, directly or indirectly related to each other. Hence, the distribution of co-occurrences of these genes could be a good estimator of the function that describes the genes interdependencies. Of course, far from the ideal, the compilation of this list is also carrying a large amount of noise among the real signal. Nonetheless, we expect that the noise will contribute with a small amount or no information to the scoring function. Indeed, we will show that with this method we are able to identify and select the most significant links from the background and that these have a very relevant biological meaning.

Prior to the computation of the bi-dimensional frequency matrix, we make a discrete approximation of the values of expressions. The observed values of *x*_*k *_were discretized into rectangular bins with size *δ *defined according to Scott's rule [18]:

(3)

where *σ *is the sample standard deviation and *N *is the number of samples in the data set. It is worth mentioning that the method is not sensitive to the choice of the size of the bins and the outcome of the analysis is quite conserved for a wide range of bin sizes and/or shapes (data not shown).

**Figure 1:**
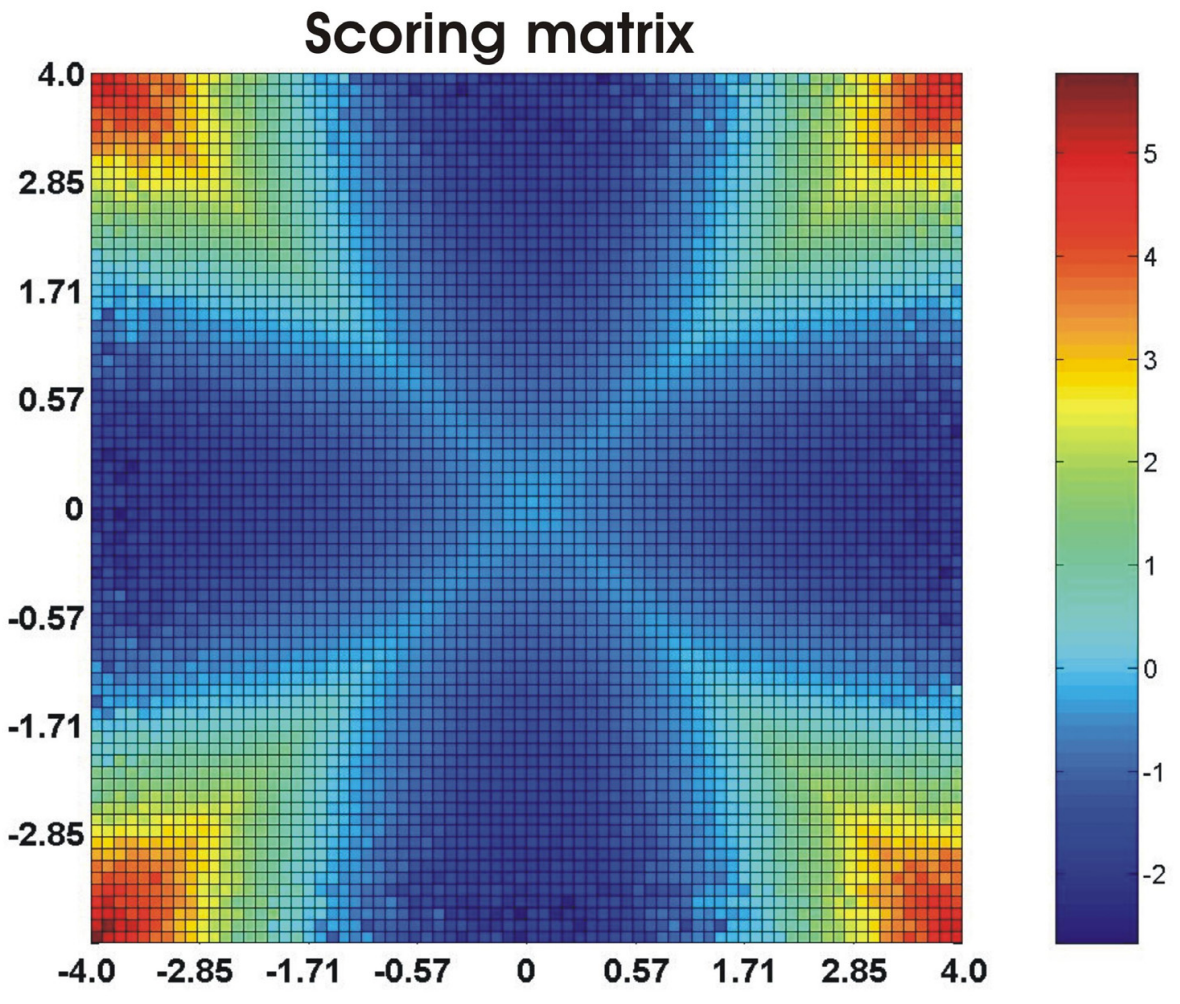
**Scoring matrix**. The distribution of log-scores *s *for Gaussian correlated variables. The artificial set was composed of series (*N *= 8 × 10^6^, *ρ *= 3) assigned in pairs with correlation value randomly chosen from an uniform distribution in [1; 1]. The number of rectangular bins to compute the frequency matrix is calculated according to Scott's rule [18].

Figure 1 shows the form of the function *s*(*x*; *y*) for an artificial set of random Gaussian correlated variables. The *x*- and *y*-axis are the bins' representative values. A high positive value of *s *(red colors) indicates that the pair (*x*; *y*) is likely to be strongly dependent on each other. The function highlights the variables lying in the diagonals (high correlation) and in the extremities of the distribution. On the other hand, log-ratios around zero contributes with zero information to the similarity score. However we would like to stress that a high measure of correlation (or dependency) between biological signals does not imply a causal relationship, hence any two given connected nodes in the networks generated from this approach are not guaranteed to be functionally related. In spite of this, our approach is a strong starting point for biological inferences.

We have proposed a method based on a standard log-likelihood score to infer association between gene expression profiles. The innovative in this work is how we calculate the *h *densities using different series of experiments altogether as a model of associated variables. Furthermore, although we use information on various sets of experiments to construct the scoring matrix, our method allows the analysis of each set individually since the similarity between pairs of genes can be assessed summing over the scores for any subset of the entire data set. Given the similarity matrix relating the *N *genes, one must set a cutoff value *S*_0 _above which the pairs of genes are considered connected in the network.

### Removing edges originating from indirect interactions

Focusing on our objective of infering subnetworks of strongly dependent genes, the process described above is subjected to an important limitation since genes separated by one or few intermediates and thus not directly interacting may achieve a high similarity value. In fact, this procedure originates very dense networks with a high occurrence of large cliques (groups of nodes completely interconnected) that does not agree with the expected structure of cellular networks [3]. To circumvent this drawback, we perform an additional step in our pipeline that removes potential indirect interactions using a known information theoretic property [19].

The procedure to remove edges originating from indirect interactions has already been used in gene co-expression networks [9,20] and was adapted here to our purposes. It states that if both genes (*i*, *j*) and (*j*, *k*) are directly interacting and (*i*, *k*) are indirectly interacting through *j*, then *S*^*ik *^≤ *S*^*ij *^and *S*^*ik *^≤ *S*^*jk*^. Thus we search for all the triangle loops in the network and discard the edges that satisfies the inequality. To account for innacurate estimates of the difference between close values of *S *we introduce a tolerance threshold:

(4)

We acknowledge that the above restriction eliminates most of the indirect interactions at the expenses of eliminating also authentic direct interactions. Nevertheless, our main concern is to minimize the occurrence of false positives given the statistical constraints. The occurrence of false negatives, *i. e.*, the absence of an interaction that actually exists in the co-regulated gene network reduces the potentiality of the method to infer new hypotheses about the system but, contrarily to the occurrence of false positives, it would hardly imply in unnecessary expenditures of time and resources to the involved laboratory.

In the following sections, we present results for a benchmark data set obtained from Spellman [17]. This set comprises data of DNA microarrays from yeast cultures synchronized by four independent methods, which are referred to as perturbations: *α*-factor arrest, elutriation and arrest of a *cdc*15 and a *cdc*28 temperature-sensitive mutants. Since our main objective is to evaluate a new method of exploring microarray data, a long discussion of biological results is beyond our purposes. Thus, we restrict our discussions to the results obtained with the *alpha*-factor and the *cdc*15 sets. Similar results were observed for elutriation and *cdc*28 temperature-sensitive mutant sets.

## Results and Discussion

*S. cerevisiae *is one of the most studied organisms to date and there is a great amount of data about gene-to-gene and protein-to-protein interactions already validated on the web (for a review, see Saccharomyces Genome Database – ). The Spellman data set [17,21] is being widely used with the aim of validating novel gene expression data exploratory methodologies. It consists of time samples taken in four different ways of synchronizing the cell cycle. After releasing the cultures from the stimuli, samples were taken over time totalizing 73 samples: *alpha *(with 18 samples collected every 7 minutes), *cdc*15 (24 samples collected every 10 or 20 minutes), *cdc*28 (17 samples collected every 10 minutes) and elutriation (14 samples every 30 minutes). In order to recreate the conditions met in the experiments targeted by this study, we selected 12 samples – 3 time points from each experiment – according to Table 1 and all the calculations were performed over this adapted set.

**Table 1:** Samples used in this work based on the Spellman dataset [17].

**Experiments**				
	**alpha**	**cdc15**	**cdc28**	**elutriation**

**Points sampled:**	28, 63 and 98 min	30, 150 and 270 min	30, 90 and 150 min	120, 240 and 360 min
**Original dataset:**	0 to 119 min (2 cycles)	10 to 290 min (3 cycles)	0 to 160 min (2 cycles)	0 to 390 min (1 cycle)

First row are the samples chosen for the construction of the *Saccharomyces *bi-dimensional frequency matrix. Second and last rows correspond to the time range comprised by the original dataset and the number of cycles comprised by the time intervals as given by the author.

**Figure 2:**
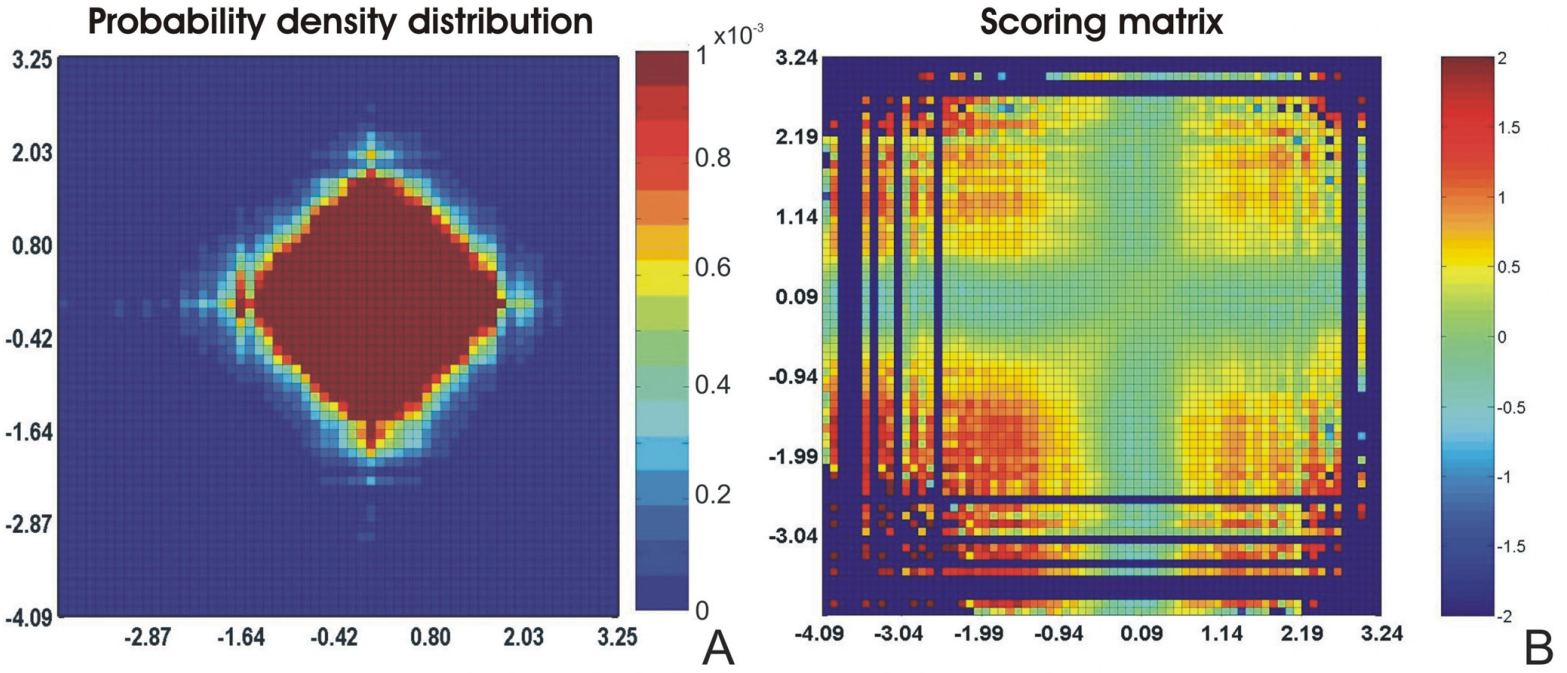
**Probability density distribution and scoring matrix for the yeast data**. The joint probability density distribution *h*(*x*; *y*) of the yeast data set (panel **A**) and the respective distribution of log-scores *s *(panel **B**). *x*- and *y*-axes are log-ratios. The intensities of the bivariate functions are given in colors. Hot colors represent higher values as scaled in the colorbars on the right. In this study, *N *= 4461 and *ρ *= 12. Genes were considered modulated if *x*_*k *_> |0.5| in at least one experimental condition.

The joint probability density distribution and the distribution of scores *s *obtained for the yeast data set are shown in Figures 2A and 2B, respectively. Intensities are represented in colors in both figures where red colors represent higher intensities. The scale is given in the colorbar on the right of each figure.

**Figure 3:**
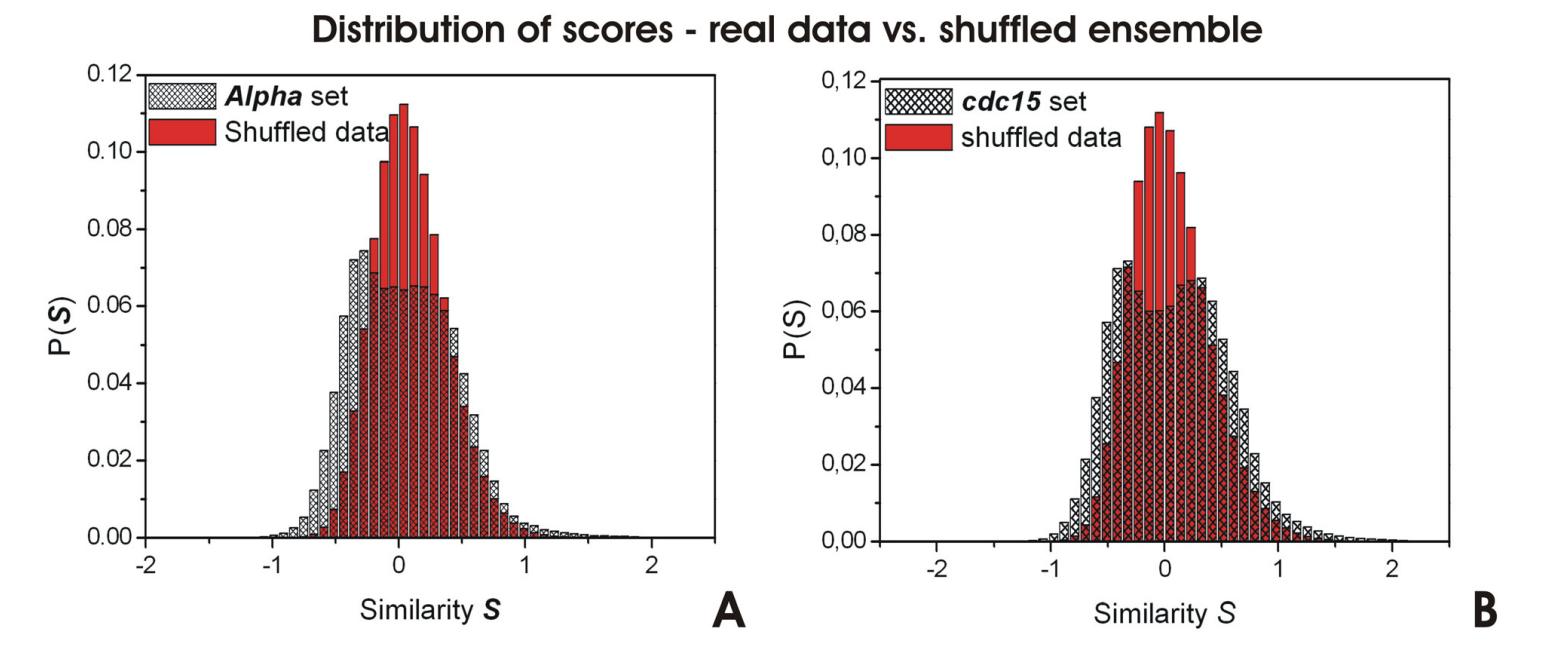
**Distribution of scores – real data vs. shuffled ensemble**. Distribution of similarity values *S *for the *alpha *and *cdc*15 data sets (shaded histogram) and their respective shuffled set (red filled histogram) representing the null model. *alpha *set – panel **A**; *cdc*15 set – panel **B**. The *alpha *set comprises 734 genes and the *cdc*15 set comprises 2517 genes modulated in at least one of *ρ' *= 3 experimental conditions.

The function *S *defined in Equation 1 was applied separately to each of the four perturbation sets and similarity matrices were obtained for each one. The distribution of *S *values obtained for the *alpha *set and the *cdc*15 set are shown in Figures 3A and 3B, respectively. The distribution of scores *S *for a set of correlated variables presents a wide range of variation and the definition of a suitable cut-off above which two nodes are said to be significantly similar is quite arbitrary. Even for uncorrelated random variables, the distribution of *S *can achieve high positive values and thus, one must pay extra care in order to define a suitable cut-off value *S*_0_. To deal with this, we generated an ensemble of uncorrelated signals by randomly shuffling the data set and averaged the obtained *S *distribution for 1000 repetitions. We then set *S*_0 _according to a value of *p *< 10^-4^. The networks were compiled assigning an undirected edge to each pair of nodes with *S *>*S*_0_. The distributions of *S *for the shuffled sets are shown superimposed to the distributions of the real data in Figure 3. One can notice the remarkable difference between the real and shuffled distributions implying that the method is capturing some biological phenomena driving the expression behavior of the genes.

Upon the removal of the indirect interactions (ε = 0.1 in Equation 4), the resulting edges were investigated for validity according to validated biological data.

### Validation of results

To validate the obtained results, each interaction found for each network (the undirected edges) is searched for biological meaning in the *BioGRID *(The *Bio*logical *G*eneral *R*epository for *I*nteraction *D*atasets) and the *Yeastract *(Yeast Search for Transcriptional Regulators And Consensus Tracking) databases [22,23]. The former is a repository of the protein and genetic interactions reported to date for some model organisms including the baker yeast. The Yeastract is a curated repository of regulatory associations between transcription factors and target genes in *S. cerevisiae *based on bibliographic references. We compared the obtained edges in two ways. First, we searched for the existence of a catalogued physical or genetic interaction in the BioGRID data bank. Second, we searched in the Yeastract data bank for the existence of a common transcription factor regulating both genes in each resulting edge. Our results show a good agreement with the literature description. In the following section we show the results for the *alpha*-factor and *cdc*15 networks.

### The networks

**Figure 4:**
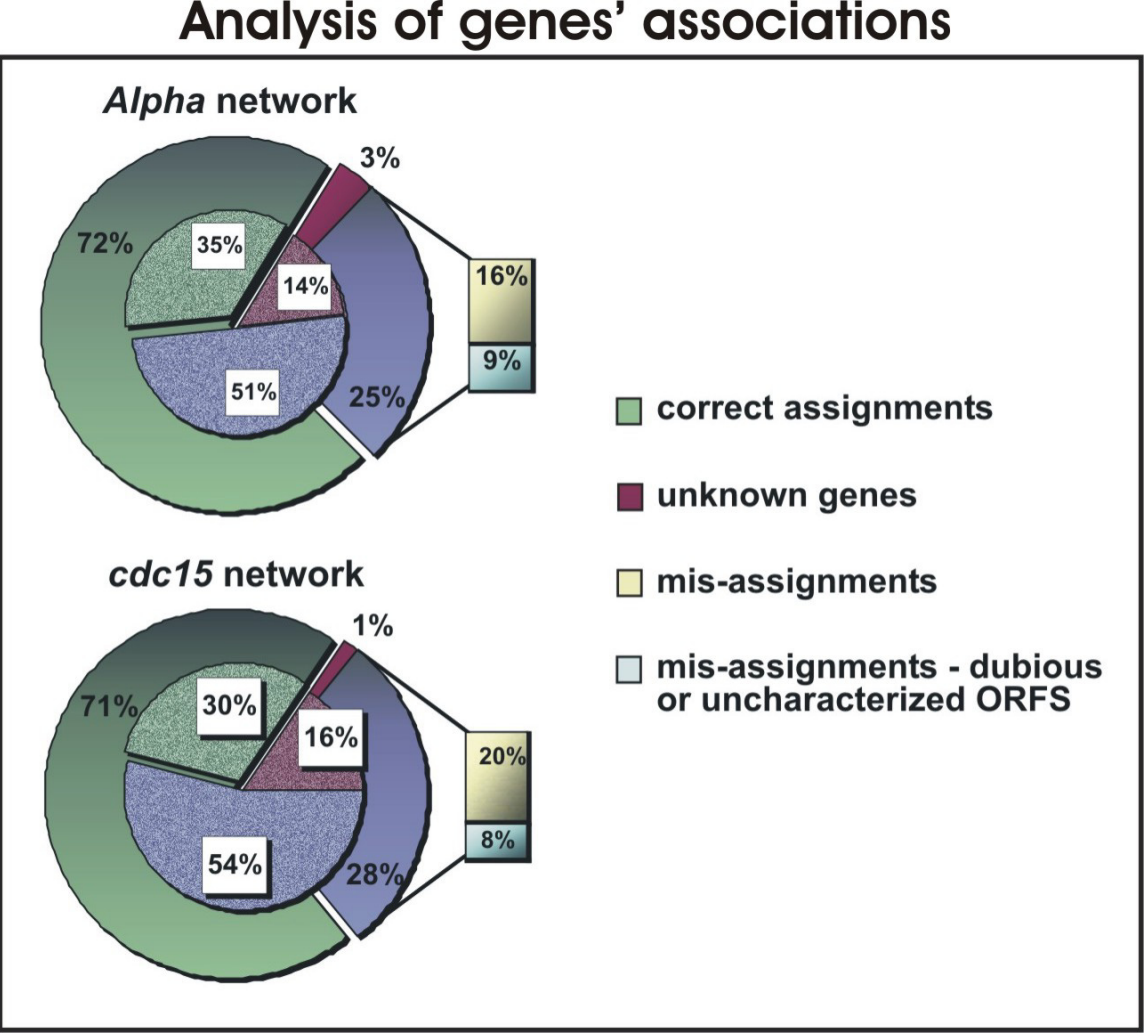
**Analysis of gene's associations**. The percentage of connections found either in the BioGRID or the Yeastract data bank (correct assignment patch) in contrast to the null model (shaded superimposed areas) for the *alpha *network and the *cdc*15 network.

The resulting edges were investigated for validity both in the BioGRID and the Yeastract data banks and results are shown in the pie charts in Figure 4. This Figure presents the percentage of connections (edges) that have either been identified in the BioGRID data bank or that are regulated by the same transcription factor as given by the Yeastract data bank. Connections involving genes not documented in the cited databases counts as unknown genes.

Connections not found in either data banks are labeled as mis-assignments and are subdivided into two groups: those that involve dubious or uncharacterized ORFs and the rest of it. In order to evaluate the results a null model has been tested by randomly assigning edges among the modulated genes while keeping the same connectivity distribution of the original network. Averaged results for this random network are shown in shaded areas.

According to these results, one can expect that in average 35% of two randomly chosen genes modulated in the *alpha *perturbation are regulated by the same transcription factor in contrast to 72% obtained with our method (*p *< 10^-5^). Similarly, in average 30% of two randomly chosen genes modulated in the *cdc*15 perturbation share a common transcription factor in contrast to 71% obtained with our method. One could argue that the rate of correct assignments for the random model is overestimated. However, we want to emphasize that the set of genes analyzed here and subjected to the described procedures comprises genes with a rate of modulation above a given threshold under the considered experimental treatment. Thus, we expect that they are all somehow related. Nonetheless, the efficiency of the method is highly above the expected by chance.

It is worth mentioning that the results shown in Figure 4 are mostly due to common documented transcription factors regulating the genes connected by an edge, *i. e.*, due to agreement with the Yeastract data bank solely. The percentage of interactions found in the BioGRID data bank is around 10% for the *alpha *network and 3% for the *cdc*15 network (*p *< 10^-5^). Although this result is statistically significant, we stress that the proposed procedure is best suited for elucidation of possible common regulation pathways rather than physical or genetic interactions as given by the BioGRID bank.

These results together reinforce the common assumption that the connections found with methods based on similarity between gene profiles are only conceptual. They mean solely that the involved pair presents patterns of expressions that are highly dependent under the statistical assumptions. Furthermore, the edges participating in the networks are selected under a rank of high statistical significance and some of them can be replaced if the parameters are changed. In spite of this, the kernel structure is usually maintained, i. e., central nodes and modular organization are usually kept under different parameter choices and can provide unparalleled valuable informations concerning genes functions and cellular states.

In order to evaluate the robustness of the method, we applied the same procedure to the complete data set (73 time points). We compared the results obtained with the complete sets of *alpha *and *cdc*15 (18 and 24 time points, respectively) to the results previously obtained (with only three time points). We observed a massive overlap between the nodes from both networks – 88% of the nodes in the *alpha *network and 97% of the nodes in the *cdc*15 network. Concerning the edges, we observed that about 10% are present in both networks. It is worth stressing that this is not a trivial result since the genes selected as nodes in the networks comprehend only a fraction of the total number of modulated genes.

### The alpha-factor network

**Figure 5:**
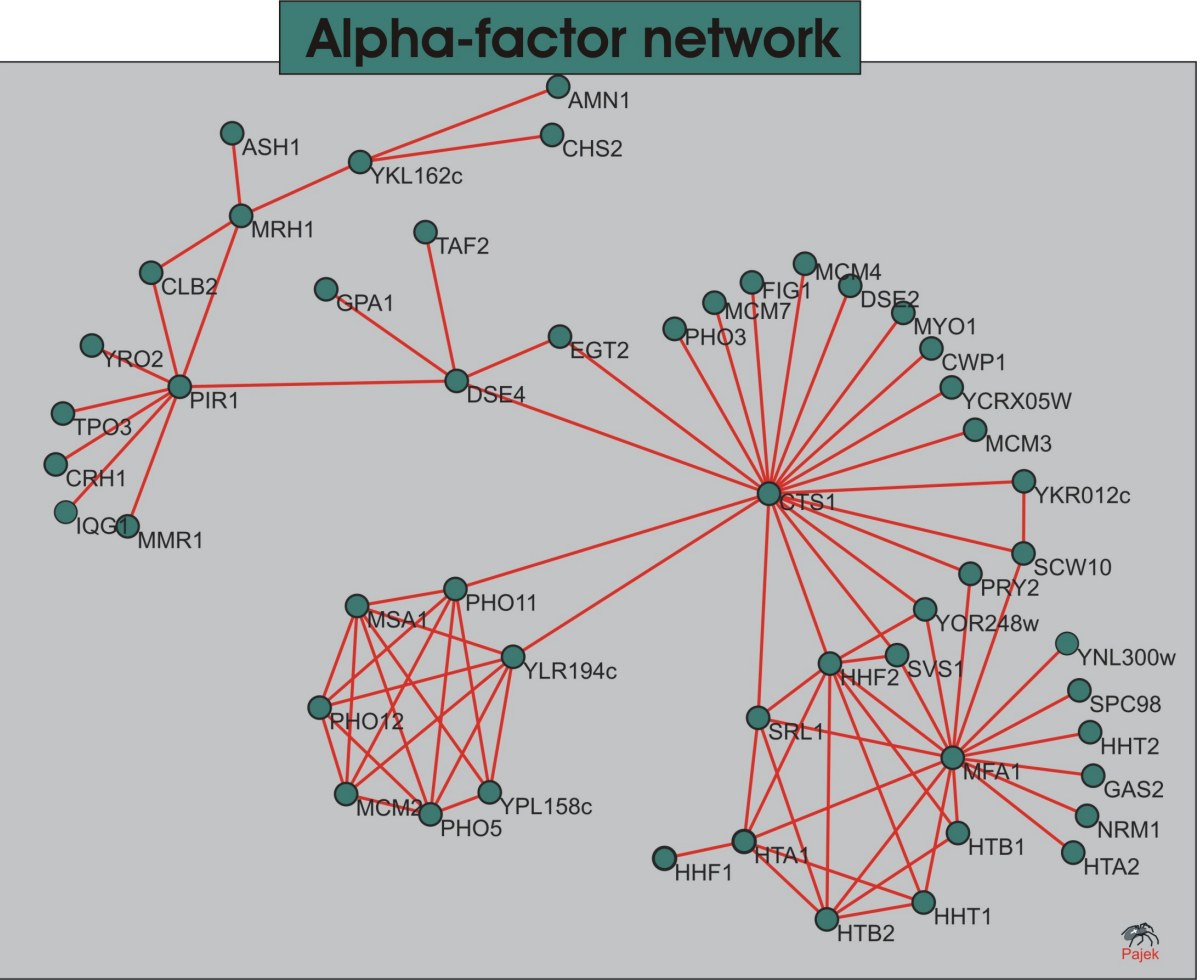
**Alpha-factor network**. The *alpha *network. The visualization of networks was performed using the Pajek program for large network analysis [40] with Fruchterman-Reingold graph layout algorithm followed by manual vertex adjustments. The identification of the genes and their description are given in Additional file 1.

The *alpha *network is depicted in Figure 5. The two most connected genes *CS T*1 and *MFA*1 encode endochitinase (required for cell separation after mitosis, activated during late GAP (**G**) and early mitosis (**M**) cell cycle phases) and Mating pheromone a-factor (which interacts with alpha cells to induce cell cycle arrest and other responses leading to mating), respectively. A list with all the genes and its annotations according to the GEO is given as Supplementary file.

**Figure 6:**
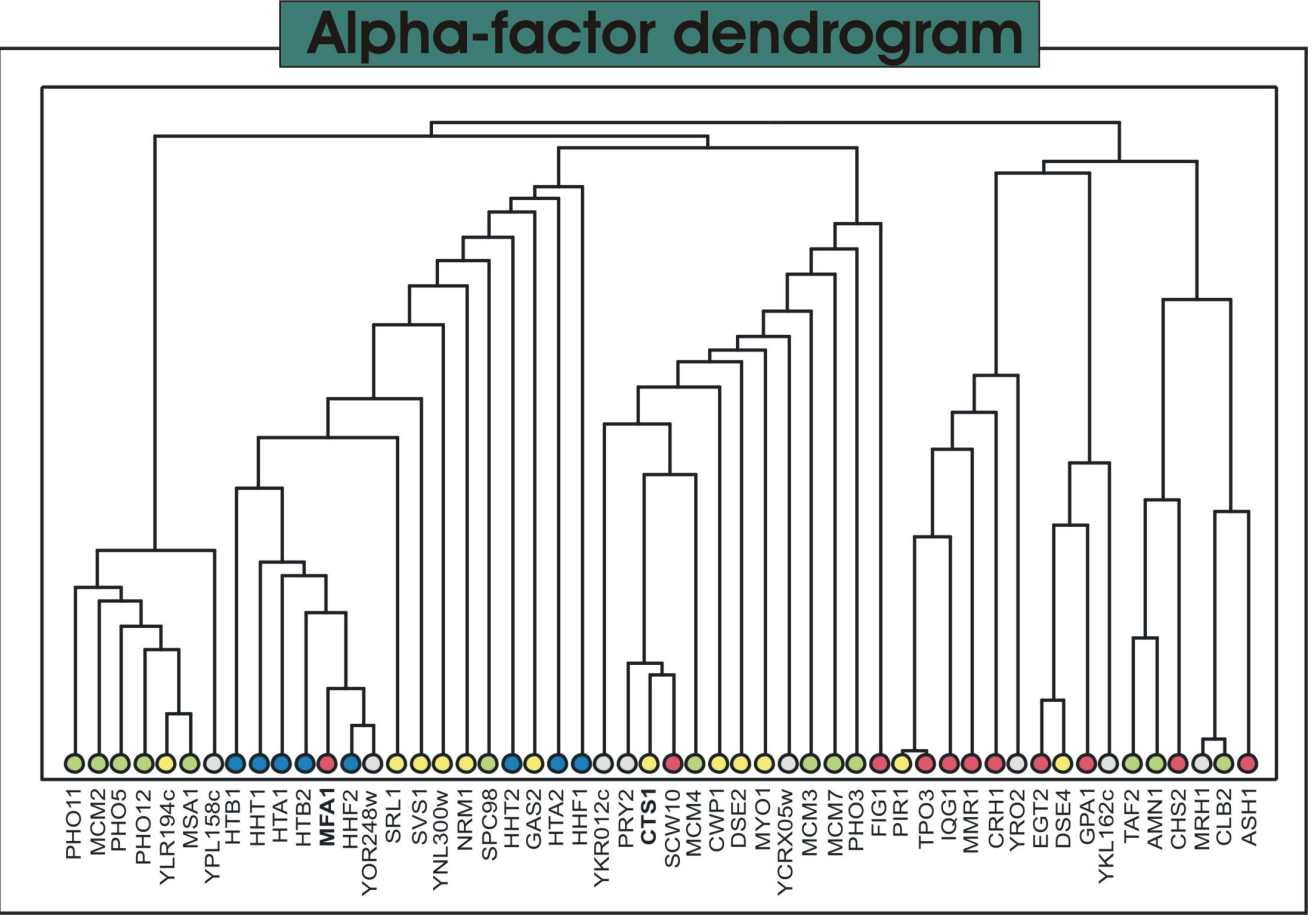
**Alpha-factor dendrogram**. The dendrogram resulted from the edge betweenness community detector applied on the *alpha *network. The colors of the leaf nodes correspond to the described protein functions: **Red**: Mating – Proteins directly or indirectly involved in cell-type-specific transcription and pheromone response; **Yellow**: Cell wall – Proteins related to cell wall remodeling. Cell-cycle regulated; **Blue**: Chromatin – Proteins required for chromatin assembly and chromossome function; **Light Green**: Cell cycle – Proteins involved in regulation of cell cycle progression; **Grey**: Unknown-Uncharacterized ORFs or proteins with unknown biological function. The edge betweenness algorithm was performed with the igraph R package [41].

Since interactions are only conceptual we suggest that instead of analyzing individual genes and edges in an isolated way, one could extract more valuable information from the analysis of groups of densely connected genes. The symbolic representation of a network in groups or modules can help one to better comprehend its structure. In Figure 5 one can nearly distinguish four groups of nodes. To facilitate the evaluation of the modules, we applied a well-known community detection procedure based on edge betweenness over the network [24,25]. The edge betweenness community algorithm splits apart modules of nodes densely connected by successively removing the most central edges. This method produces a dendrogram displaying the nodes in the *x*-axis and the distance among them is proportional to the length of the *y*-axis. The dendrogram relative to the *alpha*-factor network is given in Figure 6.

Spellman experiment's main objective was to investigate cell-cycle regulated genes [17,21]. With this aim, the authors synchronized yeast cultures in different ways analyzing transcript levels as a function of time after the release of the cultures. Each different method used to synchronize cells introduces characteristic artifacts that can persist for some time even after the stimulus is removed. Specifically, this network is originated from the use of the *α *pheromone to arrest *MATa *cells in *G*1. Yeast have two mating types, **a **and *α *(genotypes *MATa *and *MATα*, respectively) that can fuse to form a diploid *MATa*/*MATα*. Once a *MATa *cell is exposed to *α*-factor (the pheromone purified from the opposite mating type), the cell undergo a reversible process of differentiation of vegetatively growing cells to cells with characteristics of gametes. The cell ceases dividing and starts elongating towards the highest concentration of pheromone, forming a structure termed mating projection [26]. Yeast cells are non-motile; they have rigid cell wall and can't form filopodia like some protozoans. Thus, this chemotropic morphogenesis involves a series of cell wall modifications. Proteins involved in signaling, polarization, cell adhesion and fusion are localized to the mating projection [27,28]. On the other hand, in the yeast response pathway negative feedback loops operate at many levels to promote desensitization/adaptation and recovery. Among those negative feedback mechanisms, phosphorylation and dephosphorylation play crucial role in the modulation of signal intensity. Phosphatases and kinases operate at every level of the pathway.

The analysis of the *alpha *network reveals very interesting characteristics of the process going on with the studied organism. Firstly, the great majority of the genes are related to cell cycle in some way, as expected. Notwithstanding, one can discern distinct functions in different modules and most of these are directly related to the response to the pheromone, e.g. mating and cell wall remodeling. It is remarkable the presence of the clique composed by the proteins *PHO*5, *PHO*11 and *PHO*12. This module is formed by cell-cycle regulated transcripts peaking in the transition **M/G1 **of the cell cycle (Mitosis to the GAP1 phase of interphase). The acid phosphatases are involved in the response to phosphate starvation that occurs late in the cell cycle [29] due to the consumption of inorganic phosphate for the synthesis of nucleotid and phospholipids during the metaphase. The *MCM*2 and *MS A*1 are both involved in **G1/S **(GAP1/DNA Synthesis) transition progression of the cell cycle [17].

The histones module comprehends mainly transcripts peaking at the early stages of cell cycle such as the *MFA*1, *S VS *1 and *S PC*98 peaking in **G1 **and the histones themselves peaking in **S **phase. The histones play a crucial role in DNA replication and are thus highly synthesized during **S**. The *MFA*1 is the pheromone produced by **a **cells in response to mating stimulus. In our sorted time samples, it is expressed only in the very first point of the signal, maybe due to the recent stimulation with alpha-factor.

The group of genes displayed in the leftmost part of Figure 4 presents an enrichment of genes involved directly or indirectly with the cell's rearrangements in response to mating. Most of these genes peak in **M **or in the transition **M/G1**. It's worth stressing that such proteins are not present in any of the other studied networks. This module is connected to the other modules in the network through an endochitinase *CTS *1 that is strongly cell-cycle regulated and plays a role in cytokinesis. It is also interesting to notice that this central node connects all modules through at least one transcript related to cell wall assembly or remodeling.

These results show a high level of coherence in face of the known biological processes. The subnetwork of most dependent genes modulated at the *alpha*-factor stimulated cell-cycle arrest shows groups of more interdependent transcripts that whether participate in a common well-defined process or happens to be expressed in the same stages of the cell-cycle. The method reveals clusters of genes cell-cycle-related or function-related and corroborate the results found in the literature.

### The cdc15 network

**Figure 7:**
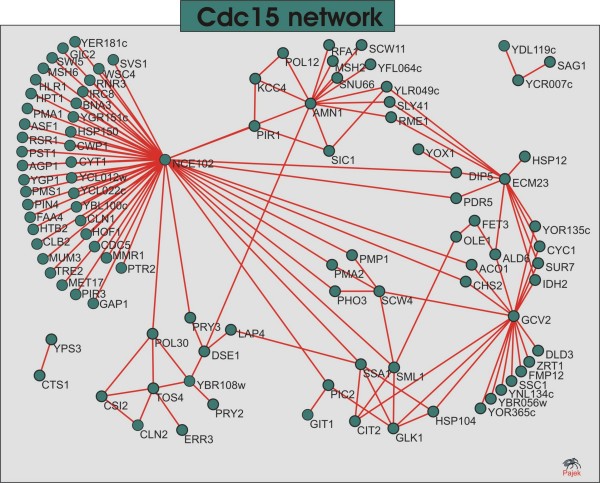
**Cdc15 network**. The *cdc*15 network. The identification of the genes and their description are given in Additional file 1.

**Figure 8:**
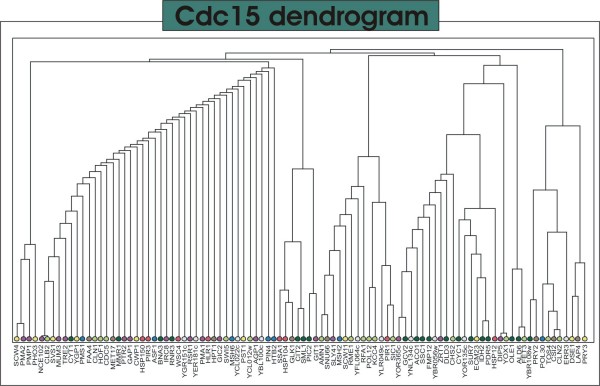
**Cdc15 dendrogram**. The dendrogram resulting from the community detection applied to the *cdc*15 network. The colours of the leaf nodes correspond to the described protein functions: **Red**: Response to stress – Proteins involved in stress response; **Yellow**: Cell Wall – Proteins related to cell wall remodeling (cell-cycle regulated); **Violet**: Membrane – Membrane integral proteins; **Green**: Mitochondria – Mitochondrial proteins; **Blue**: Chromatin – Proteins required for chromatin assembly and chromossome function; **Light Green**: Cell Cycle – Proteins involved in regulation of cell cycle progression; **Brown**: Other – several unshared biological functions; **Grey**: Unknown – Uncharacterized ORFs or proteins with unknown biological function.

The *cdc*15 subnetwork is depicted in Figure 7. In contrast to the *alpha *network, the *cdc*15 net is much denser and modules are more interconnected. The top connected vertice is *NCE*102 – a protein of unknown function, involved in secretion of proteins that lack classical secretory signal sequences; component of the detergent-insoluble glycolipid-enriched complexes (DIGs). According to the dendrogram in Figure 8, one can distinguish 6 modules in the network.

In this experiment, the authors used a temperature-sensitive mutant strain. Temperature sensitive mutants are able to grow and develop normally under a range of temperature called *permissive *but if the temperature is shifted to a so-called *restrictive *range, the organism develops the mutant phenotype and triggers a series of modifications (driven by the stress response pathways) among which is a transient cell cycle arrest. In this experiment, the authors took advantage of this genetic trait to synchronize cultures growing cells under the restrictive temperature. This procedure, however can trigger some other effects such as the stress responses induced by heat. Heat damages cells in a number of ways, perhaps most critically by disrupting the integrity of membranes and by causing proteins to denature and aggregate [30]. In response, cells induce a number of changes involving membrane fluidity and structure, an increased turnover of several plasma membrane proteins and induction of sphingolipid biosynthesis. Another characteristic feature is the strong induction of a small number of heat-shock proteins (HSPs). Their function range from the synthesis of the dissacharide trehalose, which acts as a thermoprotectant, to protein chaperones involved in protein folding, and the machinery involved in protein degradation, in particular ubiquitin [27,31,32]. Among these, the Hsp104 protein plays the most important role in yeast recovery after heat-shock exposure and a number of studies shows evidence of a connection between Hsp104 content and mitochondrial activity [33]. Mitochondria are the main source of energy in cell. They are necessary not only for growth and development, but also for the repair of heat shock induced injury. Apart from the function of the mitochondrion in energy supply, this organelle seems to regulate the expression of the HSP104 gene and probably the expression of other heat shock-regulated genes in *S. cerevisiae *[33]. Evidences suggest that the hyperpolarization of the inner mitochondrial membrane by a mild heat shock is one of several signals triggering the chain of reactions that culminates in the responses described above. In the *cdc*15 network one can notice the occurrence of several heat shock proteins including the Hsp104. In Figures 6 and 7 one can also notice that this protein is inserted in a group of proteins related to mitochondrial functions or mitochondrial integral membrane proteins. Another interesting feature of this network is the number of plasma membrane proteins, in particular proton ATPases, permeases and transporters. Certain inducers of stress responses such as heat shock permeabilizes membranes, thereby causing pronounced disturbance to transmembrane ion gradients [34]. The maintenance of the electrochemical gradient across the plasma membrane is of vital importance for the cellular functioning. Thus, a pronounced turnover of the enzymes and carrier systems associated with the membrane is expected in response to stress. This feature is being revealed in this system's depiction.

Like in the *alpha *network, here we can also discern modules of genes related to cell cycle progression such as the module centered around *TOS *4 and its neighbor centered around *AMN*1 that are mostly composed by genes that peak in the *G*1 and *S *phases. The striking predominance of cell-cycle related genes in these modules suggest a similar role to the neighboring uncharacterized proteins.

## Conclusion

Temporal gene expression data are hard to obtain and thus the design of experiments with a large set of time samples is frequently unfeasible. Instead, the majority of experiments are performed with as few as three or four time samples usually taken for as many experimental conditions. Notwithstanding the statistical poorness of the data, researchers are used to extract important clues about the biological systems by simply applying classical clustering techniques [35,36]. The availability of a more sophisticated approach could allow one to further explore the data saving time and resources in additional experimental procedures and generating more robust hypotheses to be tested. The depiction of biological systems as graphs is becoming increasingly present in the literature. The possibility of eliminating indirect interactions and to evaluate connections among groups of genes makes this a very powerful tool. Furthermore, it has been shown that centrality measures, for instance the most connected nodes, can identify gene products that play crucial roles in the analyzed processes and that these measures does not correlate with the mean expression level [37], thus rendering unparalleled informations.

Our objective in this work is to propose a metric that makes viable more robust analyses of data where standard well-established techniques are not appropriate due to the statistical constraints. Log-likelihood scores are a common tool in the inference of correlation between time series [38] that needs no prior assumptions about the functions governing it and is very well suited for the analysis of systems where little is known about it. Here we use the information contained in different experiments about how the modulated genes behave relative to each other to derive our hypothesis of association among variables.

One point to argue in this paper is the disregard of temporal correlations. Indeed, microarray time series are known to be autoregressive and good results have been obtained with linear regression models in the context of gene networks [39-41], originating directed structures where a causal relationship is implied. However, the computational cost for this kind of approach can be restrictively high and some prior assumptions have to be made concerning the regression function and the number of variables to fit the model. In this work we are proposing a more simple yet sophisticated approach. Given the nature of our data and our purposes, we chose to disregard time correlations.

Despite the simplification, our results show a very good support from biological meanings. We stress that although our method is not based on the presence or absence of oscillatory patterns, it is applicable in cases when oscillatory patterns can be assumed negligible due to the difference in time scale with the known rhythms or asynchrony of the cell culture, for instance.

We showed results for the yeast *S. cerevisiae*, a well-known biological system and discussed two very diverse cellular processes based on the networks results. The proposed *S *score allowed us to infer subnetworks of strongly dependent genes that show great biological relevance. Although the data set used here has been constructed from a large temporal series with a good time resolution, we have designed it in a way to disassemble time correlations and to impoverish statistics to an extreme condition. Even so, we were able to explain, in some depth, non-trivial biochemical processes related to the synchronization procedures. We have also applied the same algorithm to the original complete data set and the results are stunningly robust. The networks originated from the adapted subsets seem to be samplings of the complete ones with only edges rearranged (although around 10% of the edges are conserved).

Our results showed that the co-expression networks constructed from this kind of poorly temporally resolved microarray data are not appropriate to reveal physical interactions. However, it can provide a reliable tool to infer pairs of genes regulated by common transcription factors and thus extract valuable clues about how the system works. Finally, we would like to stress that this method can be applied to arbitrarily complex organisms and does not rely on prior knowledge. Likewise, it is also well suited for the analysis of data series extracted from any complex system such as social relationships or economics.

## Authors' contributions

MGC: Conducted research, designed the study, designed the algorithm, performed literature validation, wrote manuscript. FMS and IM: Participated in design of the study, participated in literature validation. OK: Participated in the theoretical discussions. CABP: Statistical discussions and revisions, wrote manuscript. GHG: Supervised research, wrote manuscript. All authors read and approved the final manuscript.

## Supplementary Material

Additional file 1 **Annotations**. A list with the genes' names and functions obtained from the SGD (*Saccharomyces *Genomic Database) for all the genes presented in the networks described in the text. Click here for file
